# Supplementary material for: A large Canadian cohort provides insights into the genetic architecture of human hair colour
Source: Commun Biol. 2021 Nov 4;4:1253. doi: 10.1038/s42003-021-02764-0 (PMC8568909; doi:10.1038/s42003-021-02764-0)
Supplement: Supplementary file 2 — Supplementary Information [file 42003_2021_2764_MOESM2_ESM.pdf]

Supplementary Information

A large Canadian cohort provides insights into the genetic architecture of human hair colour

Authors: Frida Lona-Durazo, Marla Mendes, Rohit Thakur, Karen Funderburk, Tongwu Zhang, Michael A. Kovacs, Jiyeon Choi, Kevin M. Brown, Esteban J. Parra

Supplementary Tables

Supplementary Table 1. Number of individuals on each binary hair colour category by genotyping array. In red are the samples not considered in the GWAS due to low number of cases.

HAIR COLOUR MODELS						
**MODEL**	**Binary categories**	**Axiom 2.0 UK Biobank**	**GSA 24v1+MDP**	**GSA 24v2+MDP**	**GSA 24v1**	**Omni 2.5**
**M1**	**BLONDE (1)**	590	398	307	56	57
	**BROWN + BLACK (0)**	3981	3988	2193	378	450
**M2**	**BROWN (1)**	3805	3592	2037	343	409
	**BLACK (0)**	176	396	156	35	41
**M3**	**RED (1)**	167	72	49	2	15
	**BROWN + BLACK (0)**	3981	3988	2193	378	450

Supplementary Table 2. GCTA approximate conditional and joint analysis (COJO) results of each hair colour category. chr= chromosome; position= chromosomal position (hg19); MAF= minor allele frequency; OR= odds ratio; SE= standard error of the log(OR); FE= fixed effects model; RE2= random effects model; LD: linkage disequilibrium with the SNP in the line below; I^2= heterogeneity measure, where $I^2 > 50$ is considered as notable heterogeneity[1]; Q= Cochran's Q estimate of heterogeneity.

Blonde Hair Colour																
**chr**	**rsid**	**position**	**Ref. Allele**	**frequency**	**log(OR)**	**SE**	**p-value (RE2)**	**p-value (FE)**	**joint log(OR)**	**joint SE**	**joint p-value**	**LD (r^2)**	**Nearest Gene**	**I^2**	**Q**	**Q (p-value)**
5	rs35391	33955673	C	0.034	-0.716	0.117	1.52E-09	8.59E-10	-0.716	0.117	9.81E-10	0	*SLC45A2*	0.000	2.173	0.704
6	rs12203592	396321	T	0.835	0.897	0.059	8.89E-52	2.22E-52	0.897	0.060	4.50E-50	0	*IRF4*	0.000	2.584	0.630
11	rs72932523	68906120	A	0.900	-0.446	0.070	1.44E-10	2.07E-10	-0.446	0.070	2.41E-10	0	*TPCN2*	64.418	11.242	0.024
12	rs12821256	89328335	C	0.913	-0.466	0.072	1.64E-10	8.87E-11	-0.466	0.072	1.03E-10	0	*KITLG*	66.806	12.051	0.017
14	rs12896471	92773903	G	0.586	-0.477	0.041	3.47E-30	1.12E-30	-0.477	0.042	5.10E-30	0	*SLC24A4*	0.000	3.814	0.432
15	rs12913832	28365618	G	0.338	-1.190	0.047	3.27E-140	5.03E-141	-1.190	0.050	7.74E-123	0	*HERC2*	0.000	3.269	0.514
16	rs35063026	89736157	T	0.950	-0.741	0.097	4.62E-14	2.28E-14	-0.741	0.098	3.13E-14	0	*SPATA33*	11.006	4.495	0.343
**Brown Hair Colour**																
**chr**	**rsid**	**position**	**Ref. Allele**	**frequency**	**log(OR)**	**SE**	**p-value (RE2)**	**p-value (FE)**	**joint log(OR)**	**joint SE**	**joint p-value**	**LD (r^2)**	**Nearest Gene**	**I^2**	**Q**	**Q (p-value)**
5	rs35406	33946143	G	0.974	1.344	0.185	6.77E-13	3.31E-13	1.344	0.185	3.87E-13	0	*SLC45A2*	30.030	5.717	0.221
6	rs12203592	396321	T	0.821	1.138	0.076	6.72E-50	1.76E-50	1.138	0.077	2.67E-49	0	*IRF4*	19.368	4.961	0.291
15	rs1129038	28356859	T	0.369	-0.890	0.059	1.17E-51	3.36E-52	-0.890	0.059	5.14E-51	0	*HERC2*	40.579	5.049	0.168
**Red Hair Colour**																
**chr**	**rsid**	**position**	**Ref. Allele**	**frequency**	**log(OR)**	**SE**	**p-value (RE2)**	**p-value (FE)**	**joint log(OR)**	**joint SE**	**joint p-value**	**LD (r^2)**	**Nearest Gene**	**I^2**	**Q**	**Q (p-value)**
16	rs12931267	89818732	G	0.942	-4.245	0.221	2.00E-82	2.29E-82	-3.735	0.252	1.17E-49	0.007	*FANCA*	78.660	9.372	0.009
16	rs1805008	89986144	T	0.948	-2.369	0.218	1.99E-28	2.02E-27	-1.888	0.245	1.22E-14	0.002	*MC1R*	86.589	14.913	0.001
16	rs1805009	89986546	C	0.978	-3.633	0.373	2.32E-22	1.83E-22	-4.241	0.380	5.40E-29	0.004	*MC1R*	67.703	6.192	0.045
16	rs77901106	90015027	A	0.876	0.924	0.139	5.14E-11	3.23E-11	0.964	0.153	2.94E-10	0.090	*DEF8*	0.000	0.391	0.822
16	rs35176381	90062479	A	0.321	1.457	0.100	5.50E-48	1.69E-48	1.014	0.124	2.27E-16	0.000	*AFG3L1P*	25.620	2.689	0.261

Supplementary Table 3. Most likely candidate causal variants inferred by FINEMAP and subsequent annotation, on each hair colour model. We defined the most likely candidate causal variants based on their $\log_{10}$BF (≥ 2) and posterior annotation (See Methods for details). CS= credible set; Position= chromosomal position (hg19); MAF= minor allele frequency; PIP= posterior inclusion probability of the SNP being causal; $\log_{10}$BF= log10 Bayes Factor; FM= foreskin melanocytes; K= keratinocytes. Annotation details available on Supplementary Files 4-6 for all candidate causal SNPs with $\log_{10}$BF ≥ 2.

**Blonde Hair Colour**								
**rsid**	**CS**	**Chromosome**	**Position**	**MAF**	**Gene**	**PIP**	**$\log_{10}$BF**	**Coding / Regulatory Annotation**
rs16891982	1	5	33951693	0.050	*SLC45A2*	0.063	2.0	missense variant (Leu374Phe)
rs12203592	1	6	396321	0.164	*IRF4*	1.000	13.0	intron | open chromatin in FM
rs3829241	1	11	68855363	0.375	*TPCN2*	0.210	2.5	missense variant (Gly734Glu)
rs12913832	1	15	28365618	0.336	*HERC2*	0.979	4.4	intron | open chromatin in FM
rs1805005	1	16	89985844	0.138	*MC1R*	0.984	5.0	missense variant (Val60Leu)
rs1805007	2	16	89986117	0.052	*MC1R*	0.238	2.7	missense variant (Arg151Cys)
rs1805008	3	16	89986144	0.049	*MC1R*	0.314	2.8	missense variant (Arg160Trp)
**Brown Hair Colour**								
**rsid**		**Chromosome**	**Position**	**MAF**	**Gene**	**PIP**	**$\log_{10}$BF**	**Coding / Regulatory Annotation**
rs16891982	1	5	33951693	0.046	*SLC45A2*	0.898	4.1	missense variant (Leu374Phe)
rs12203592	1	6	396321	0.177	*IRF4*	1.000	13.0	intron | open chromatin in FM
rs12913832	1	15	28365618	0.366	*HERC2*	0.577	2.9	intron | open chromatin in FM
rs7168800	2	15	28341575	0.100	*OCA2*	0.328	2.5	intron | open chromatin in FM and K
**Red Hair Colour**								
**rsid**		**Chromosome**	**Position**	**MAF**	**Gene**	**PIP**	**$\log_{10}$BF**	**Coding / Regulatory Annotation**
rs113891247	1	16	90047757	0.057	*AFG3L1P*	0.999	6.2	intron | open chromatin in FM
rs1805009	2	16	89986546	0.021	*MC1R*	0.978	4.9	missense variant (Asp294His)
rs1805008	3	16	89986144	0.051	*MC1R*	0.602	3.4	missense variant (Arg160Trp)

Supplementary Table 4. Genes used as input in FUMA for pathway enrichment analysis.

**Gene Symbol**	
SLC45A2	*MC1R*
IRF4	*SPIRE2*
TPCN2	*TCF25*
KITLG	*AFG3L1P*
SLC24A4	*SPATA33*
OCA2	*FANCA*
HERC2	

Supplementary Table 5. Summary statistics of genome-wide hits in the hair colour (HC) meta-analyses (models: 1= blonde; 2= brown; 3= red hair colour) that are also genome-wide significant in one or more skin cancer traits and sun tanning, based on the GWAS Catalog Database. BCC: basal cell carcinoma; SCC: squamous cell carcinoma; (+) indicates significance in the hair colour GWAS and in the phenotype tested. (-) indicates significance only in the hair colour GWAS.

HC model	rsid	chr	pos	Nearest Gene	beta_FE	std_FE	pvalue_FE	BCC	SCC	Melanoma	Suntan
2	rs35407	5	33946571	*SLC45A2*	-1.23	0.19	5.45E-11	+	+	+	-
1	rs35407	5	33946571	*SLC45A2*	-0.76	0.13	8.40E-09	+	+	+	-
2	rs16891982	5	33951693	*SLC45A2*	-1.36	0.18	1.20E-14	+	+	+	+
1	rs16891982	5	33951693	*SLC45A2*	-0.72	0.12	3.50E-09	+	+	+	+
1	rs12203592	6	396321	*IRF4*	0.90	0.06	2.22E-52	+	+	-	-
2	rs12203592	6	396321	*IRF4*	1.14	0.08	1.76E-50	+	+	-	-
1	rs62389423	6	421281	*IRF4/EXOC2*	0.75	0.09	1.06E-15	-	-	+	-
2	rs62389423	6	421281	*IRF4/EXOC2*	1.19	0.15	1.03E-14	-	-	+	-
1	rs12210050	6	475489	*IRF4/EXOC2*	0.60	0.08	3.83E-15	+	-	-	-
2	rs12210050	6	475489	*IRF4/EXOC2*	0.45	0.06	6.96E-15	+	-	-	-
1	rs4904871	14	92795912	*SLC24A4*	0.45	0.05	1.98E-19	-	-	+	-
1	rs1800407	15	28230318	*OCA2*	0.51	0.10	1.64E-07	-	+	+	+
2	rs4778138	15	28335820	*OCA2*	0.75	0.06	3.31E-38	-	+	+	-
1	rs4778138	15	28335820	*OCA2*	0.59	0.07	9.53E-17	-	+	+	-
1	rs1129038	15	28356859	*HERC2*	-1.18	0.05	1.16E-139	-	-	-	+
2	rs1129038	15	28356859	*HERC2*	-0.89	0.06	3.36E-52	-	-	-	+
1	rs12913832	15	28365618	*HERC2*	-1.19	0.05	5.03E-141	-	-	+	+
2	rs12913832	15	28365618	*HERC2*	-0.90	0.06	3.40E-52	-	-	+	+
1	rs1667394	15	28530182	*HERC2*	-1.01	0.05	2.98E-77	-	-	-	-
2	rs1667394	15	28530182	*HERC2*	-0.63	0.07	3.55E-22	-	-	-	-
3	rs117156175	16	89235401	*RP11-46C24.3*	-3.61	0.34	1.35E-26	-	-	-	-
3	rs117382825	16	89262162	*CDH15/SLC22A31*	-3.57	0.34	3.68E-26	-	-	+	-
3	rs4499232	16	89576507	*SPG7*	0.89	0.09	7.27E-21	-	-	+	-
3	rs35063026	16	89736157	*SPATA33*	-3.87	0.22	1.37E-67	-	+	-	-
1	rs35063026	16	89736157	*SPATA33*	-0.74	0.10	2.28E-14	-	+	-	-
3	rs258322	16	89755903	*CDK10*	3.19	0.19	1.21E-62	-	-	+	-
1	rs258322	16	89755903	*CDK10*	0.49	0.08	1.12E-09	-	-	+	-
3	rs35026726	16	89791279	*ZNF276*	-4.28	0.23	7.81E-80	-	-	+	-
1	rs35026726	16	89791279	*ZNF276*	-0.70	0.10	5.66E-13	-	-	+	-
3	rs1805007	16	89986117	*MC1R*	-4.05	0.22	8.07E-78	+	+	+	+
1	rs1805007	16	89986117	*MC1R*	-0.72	0.10	4.14E-14	+	+	+	+
3	rs4268748	16	90026512	*DEF8*	-1.74	0.11	1.25E-51	-	+	-	-
3	rs8063761	16	90027626	*DEF8*	-1.54	0.10	2.63E-49	-	+	-	-
3	rs4785763	16	90066936	*AFG3L1P*	1.40	0.10	4.28E-44	-	-	+	-

Supplementary Table 6. Total number of samples and markers on each genotyping chip array, before and after quality control.

Genotyping Array	Genotype data before QC		Genotype data before imputation		PCA outliers	TOTAL (N)
	Samples	SNPs	Samples	SNPs	N	
**Omni 2.5**	526	2,349,746	526	2,081,743	1	525
**Axiom 2.0 UK Biobank**	4,821	813,168	4,762	630,508	17	4745
**GSA 24v1**	438	626,377	438	558,183	0	438
**GSA 24v2+MDP**	2,594	728,919	2,573	596,061	20	2553
**GSA 24v1+MDP**	4,617	658,296	4,523	600,859	43	4480
**TOTAL**	**12996**	**-**	**12822**	**-**	**81**	**12741**

Supplementary Figures

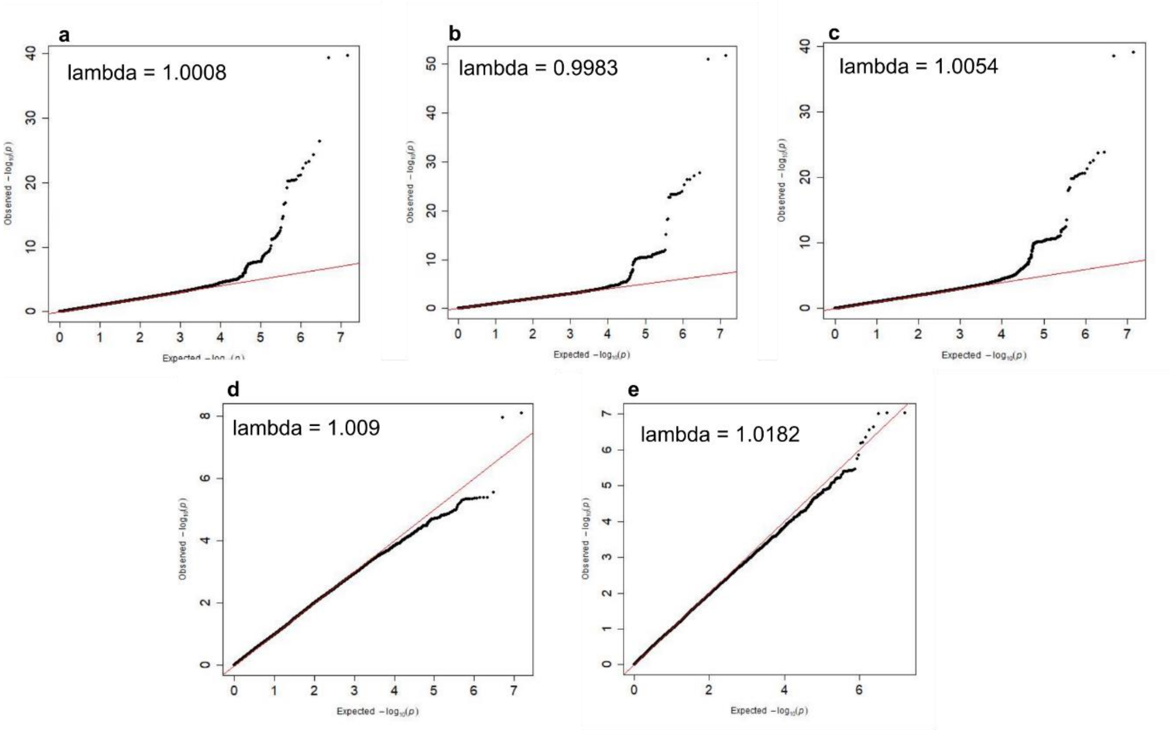

Supplementary Figure 1. Q-Q plots (-log$_{10}$ p-values) of each cohort for the GWAS blonde hair colour, showing the genomic inflation factor (lambda λ). a = Axiom 2.0 UK Biobank; b = GSA 24v1+MDP; c = GSA 24v2+MDP; d = Omni 2.5; e = GSA 24v1. The red line on each panel is a reference indicating no deviation from the expected distribution.

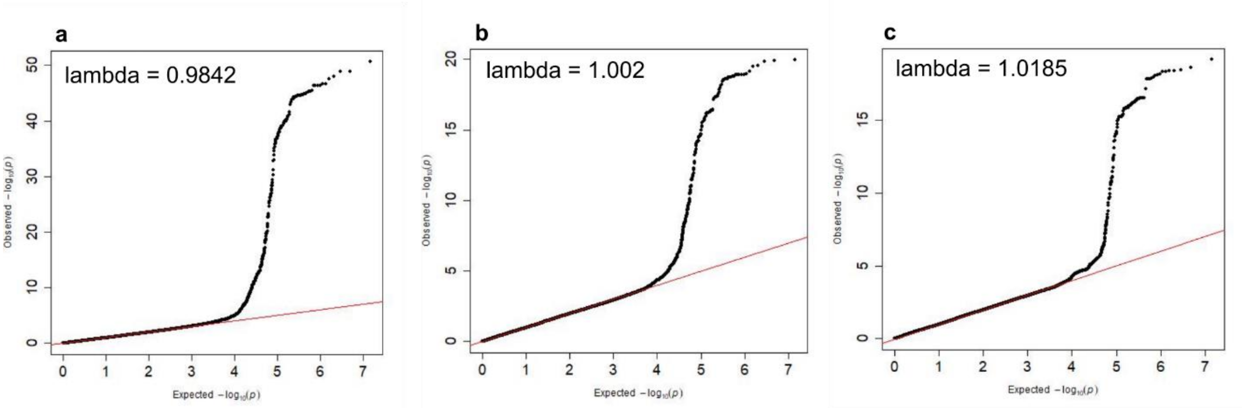

Supplementary Figure 2. Q-Q plots (-log$_{10}$ p-values) of each cohort for the GWAS red hair colour, showing the genomic inflation factor (lambda λ). a = Axiom 2.0 UK Biobank; b = GSA 24v1+MDP; c = GSA 24v2+MDP. The red line on each panel is a reference indicating no deviation from the expected distribution.

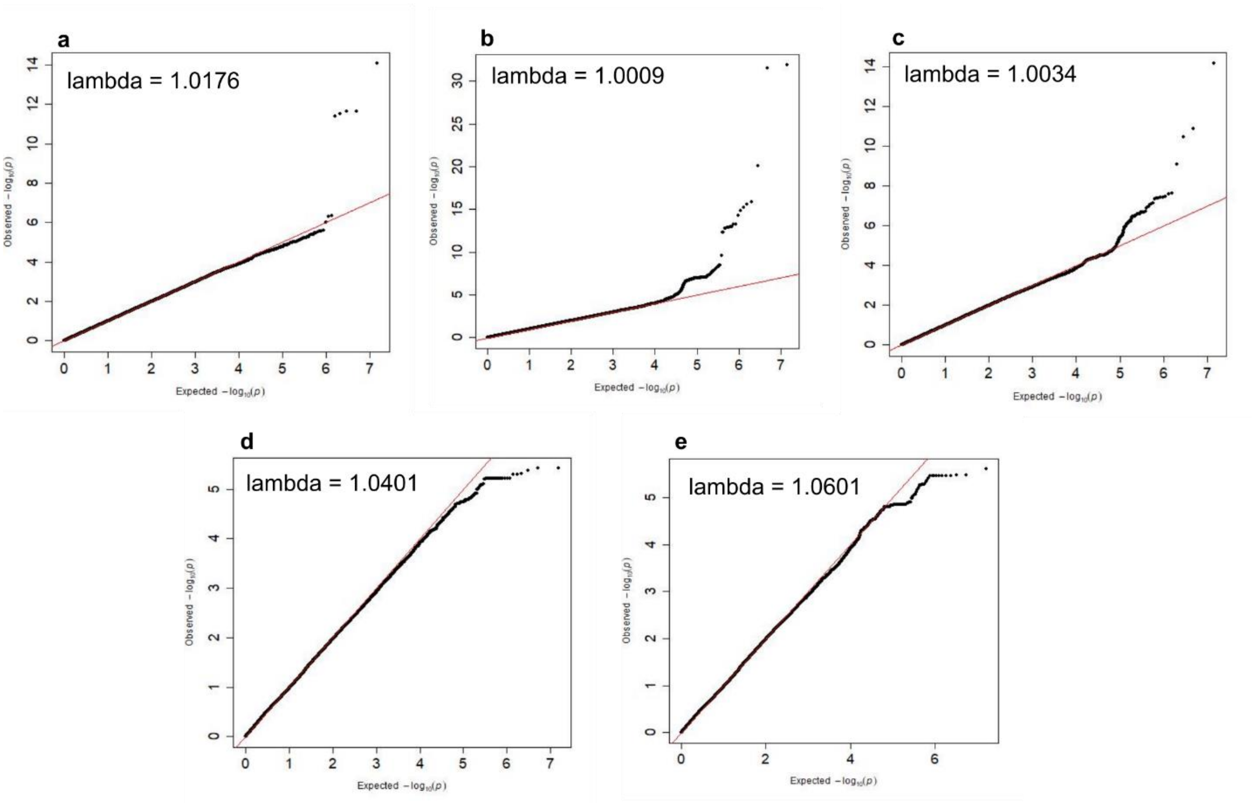

Supplementary Figure 3. Q-Q plots (-$\log_{10}$ p-values) of each cohort for the GWAS brown hair colour, showing the genomic inflation factor (lambda λ). a = Axiom 2.0 UK Biobank; b = GSA 24v1+MDP; c = GSA 24v2+MDP; d = Omni 2.5; e = GSA 24v1. The red line on each panel is a reference indicating no deviation from the expected distribution.

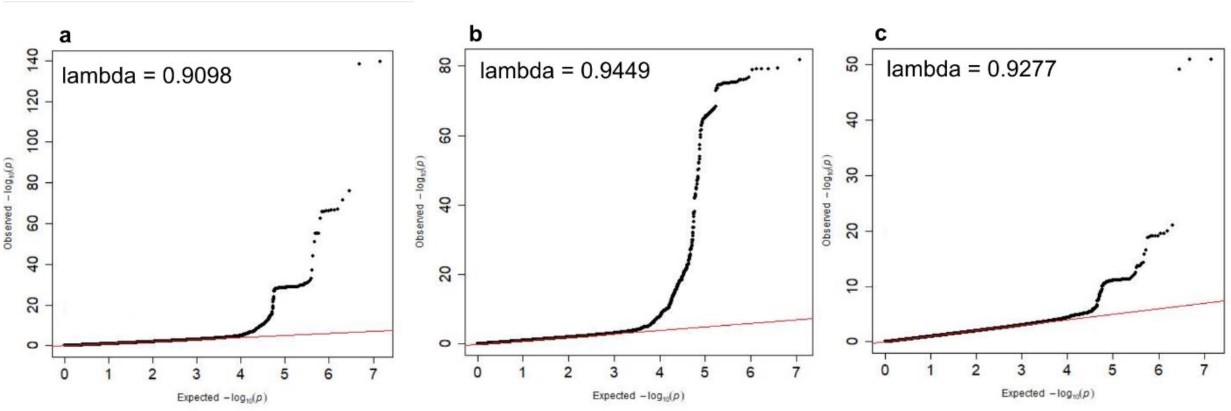

Supplementary Figure 4. Q-Q plots (-$\log_{10}$ p-values) of blonde (a), red (b) and brown (c) hair colour meta-analyses, showing the genomic inflation factor (lambda λ). The red line on each panel is a reference indicating no deviation from the expected distribution.

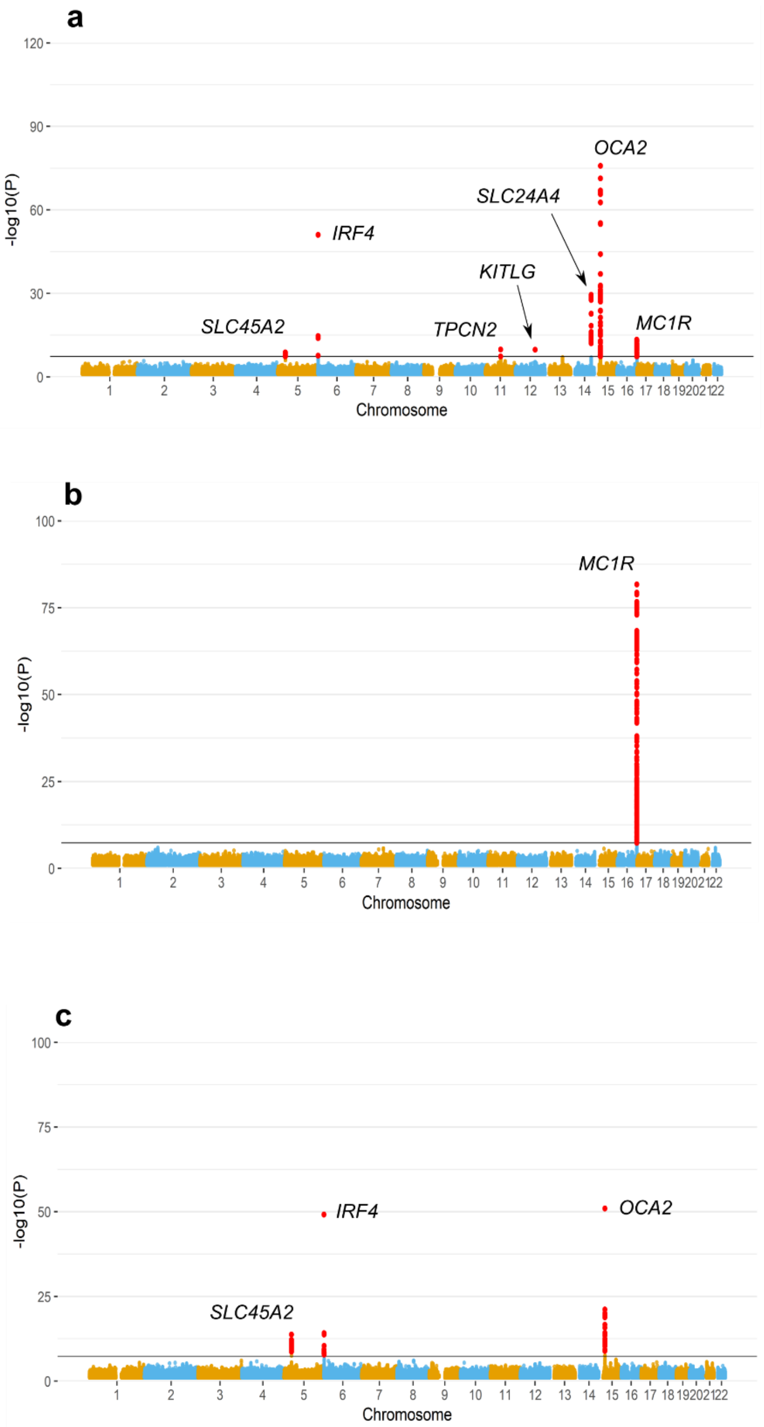

Supplementary Figure 5. Manhattan plots of hair colour meta-analyses based on logistic mixed models. a = Blonde vs. brown and black hair colour (n = 12,398 individuals). b = Red vs. brown and black hair colour (n = 10,450 individuals). c = Brown vs. black hair colour (n = 10,990 individuals). The continuous line denotes the genome-wide threshold (p= 1.67e-8). Markers in red are genome-wide significant.

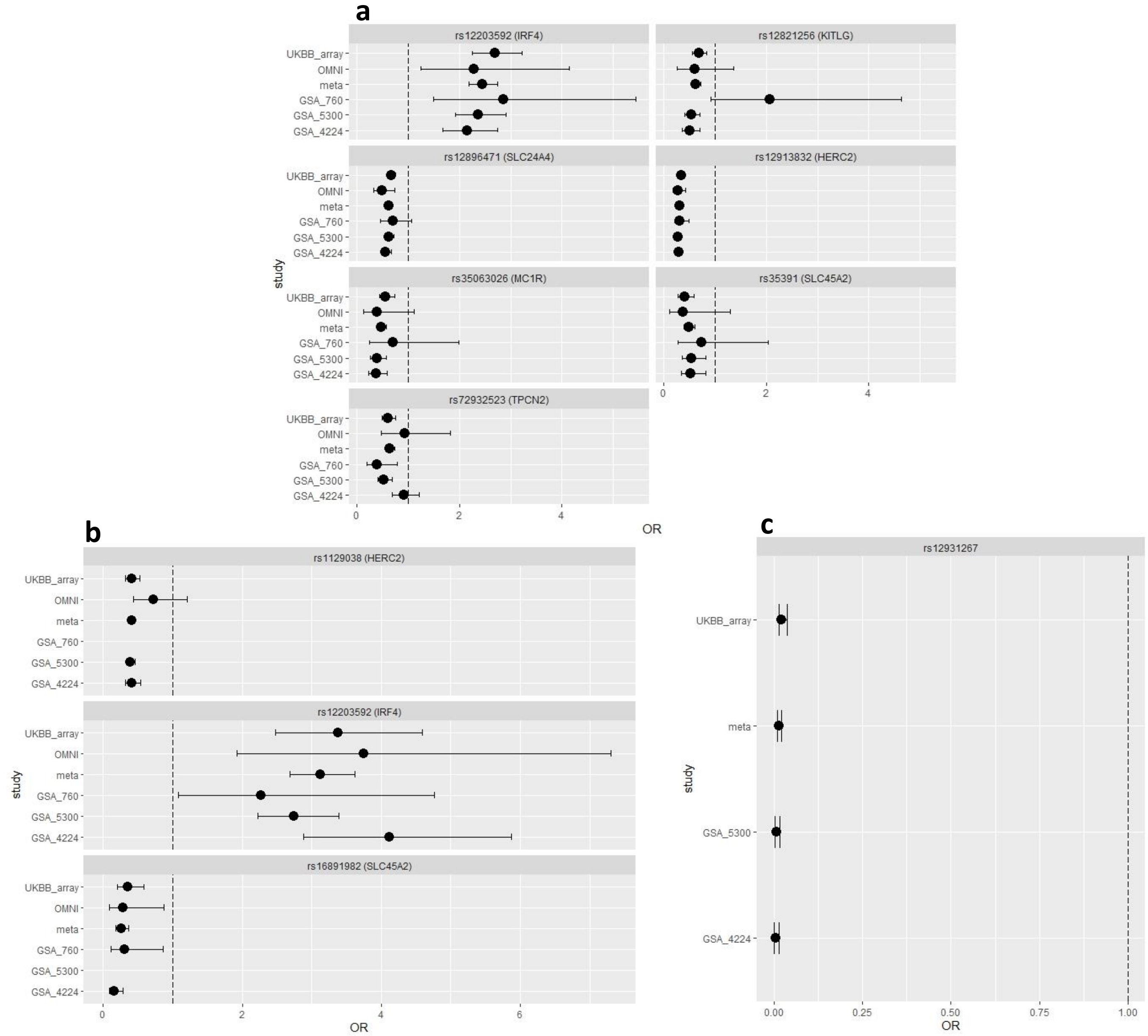

Supplementary Figure 6. Forest plots of the odds ratio (OR) of the top genome-wide significant signals in the hair colour GWAS and meta-analyses. a = Blonde vs. brown and black hair colour GWAS; b = Brown vs. black hair colour; c = Red vs. brown and black hair colour. Error bars indicate the 95% confidence intervals.

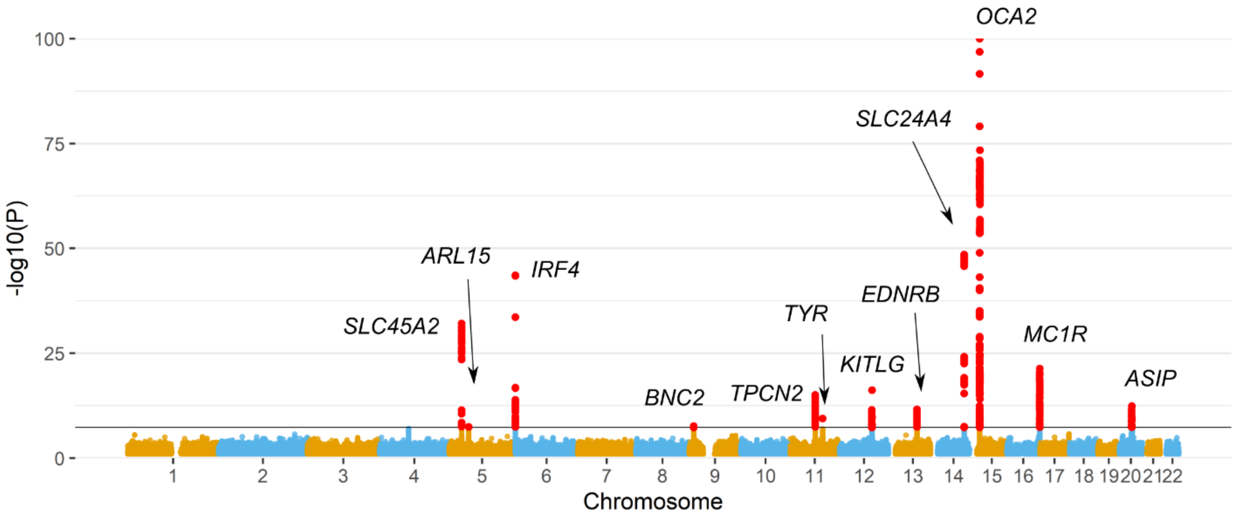

Supplementary Figure 7. Manhattan plot of the meta-analysis of hair colour performed on the CanPath cohorts using a linear model. Red datapoints are genome-wide significant ($p < 1.67e-8$) markers.

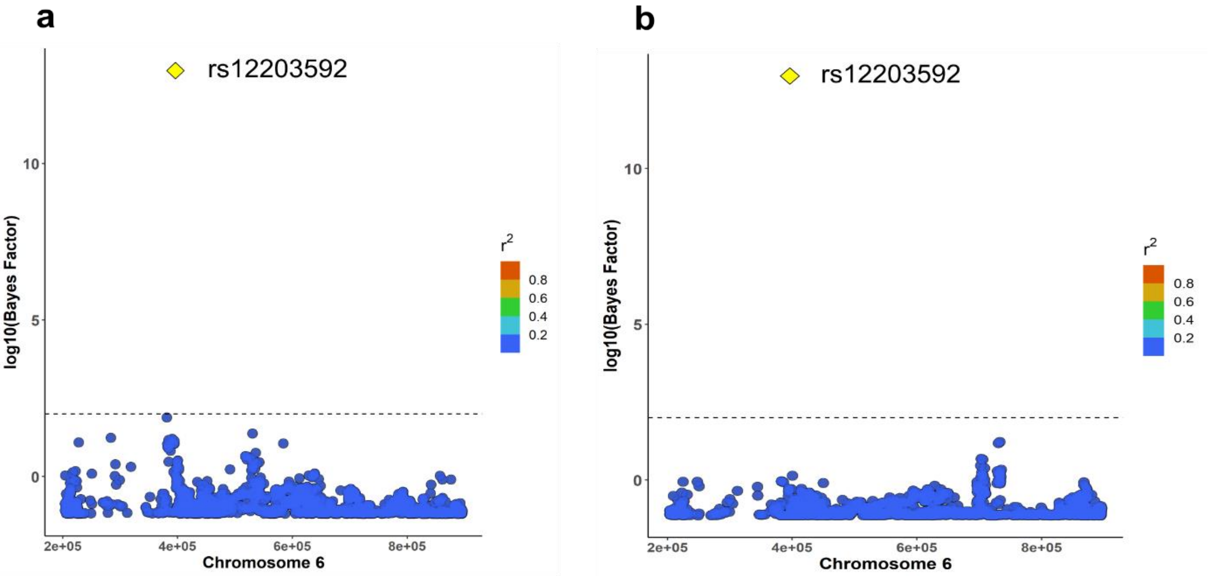

Supplementary Figure 8. FINEMAP regional plot of the *IRF4* locus (chromosome 6) associated with blonde (a) and brown (b) hair colour. The lead SNP (rs12203592) is highlighted in yellow and LD r^2 correlations are shown with respect to the lead SNP. Posterior inclusion probability (PIP) of the highlighted SNP = 1 in both cases. The dotted line is the lower threshold of the $\log_{10}$BF for considerable evidence of causality.

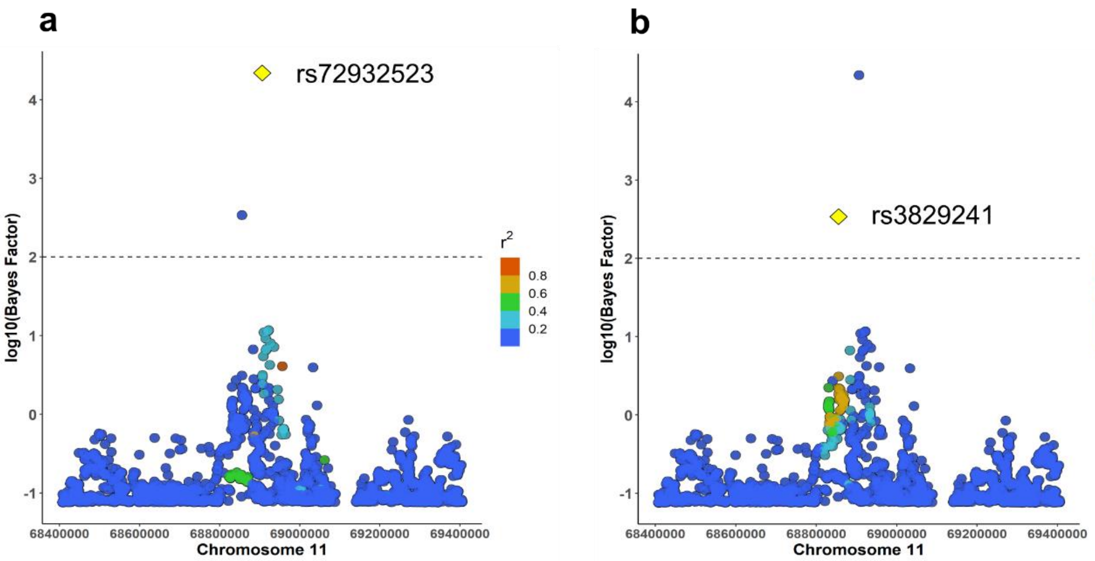

Supplementary Figure 9. FINEMAP regional plot of the *TPCN2* locus (chromosome 11) associated with blonde hair colour. LD r^2 correlations are shown with respect to the SNP highlighted in yellow (a = rs72932523; b = rs3829241). Posterior inclusion probability (PIP) of the highlighted SNPs are 0.944 and 0.210 for A and B, respectively. The dotted line is the lower threshold of the log_{10}BF for considerable evidence of causality.

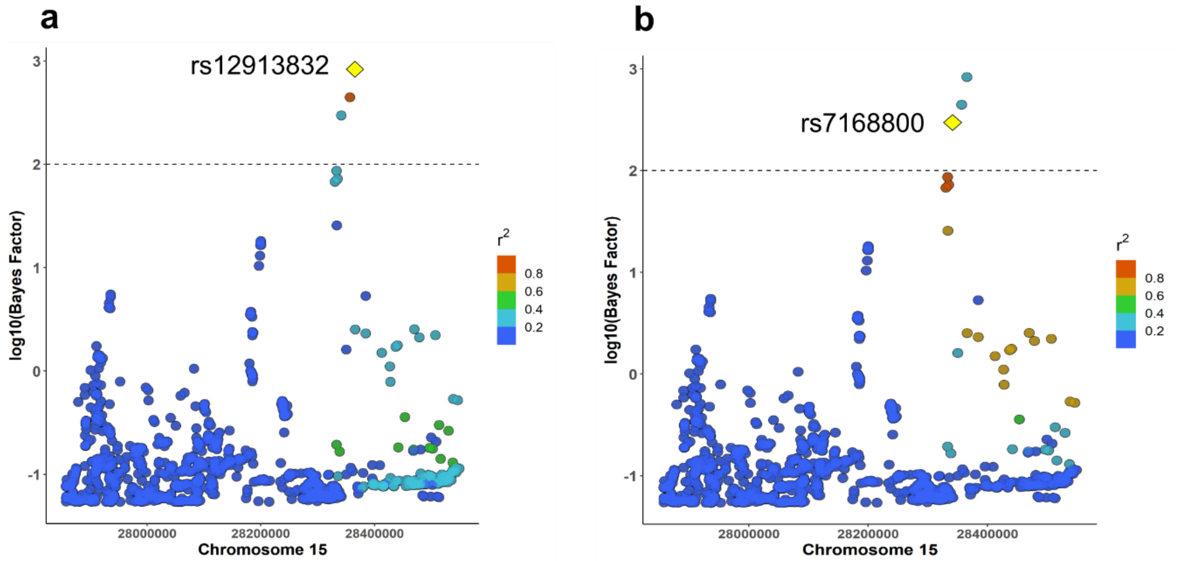

Supplementary Figure 10. FINEMAP regional plot of the *OCA2/HERC2* locus (chromosome 15) associated with brown hair colour. LD r^2 correlations are shown with respect to the SNP highlighted in yellow (a = rs12913832; b = rs7168800). Posterior inclusion probability (PIP) of the highlighted SNP are 0.577 and 0.328, respectively. The dotted line is the lower threshold of the log_{10}BF for considerable evidence of causality.

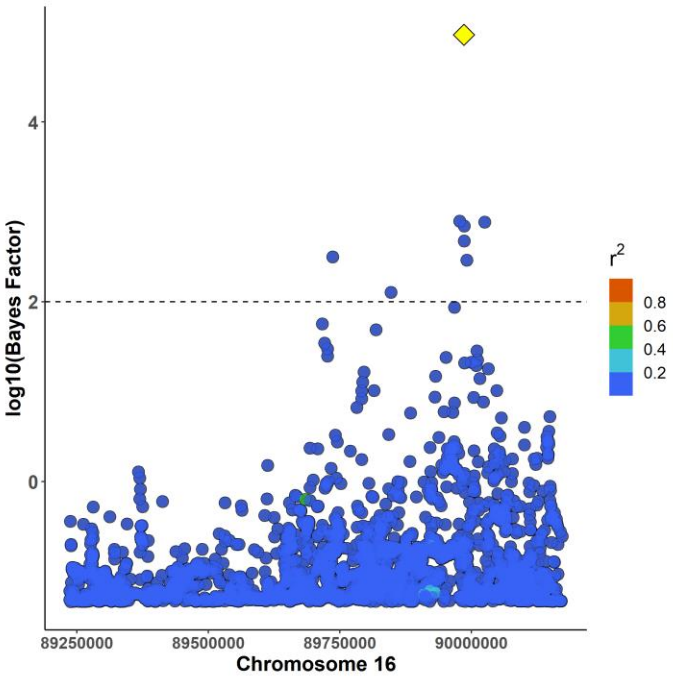

Supplementary Figure 11. FINEMAP regional plot of the *MC1R* locus (chromosome 16) associated with blonde hair colour. LD r^2 correlations are shown with respect to the SNP (rs1805005) highlighted in yellow. Posterior inclusion probability (PIP) of the highlighted SNP is 0.984. The dotted line is the lower threshold of the log_{10}BF for considerable evidence of causality.

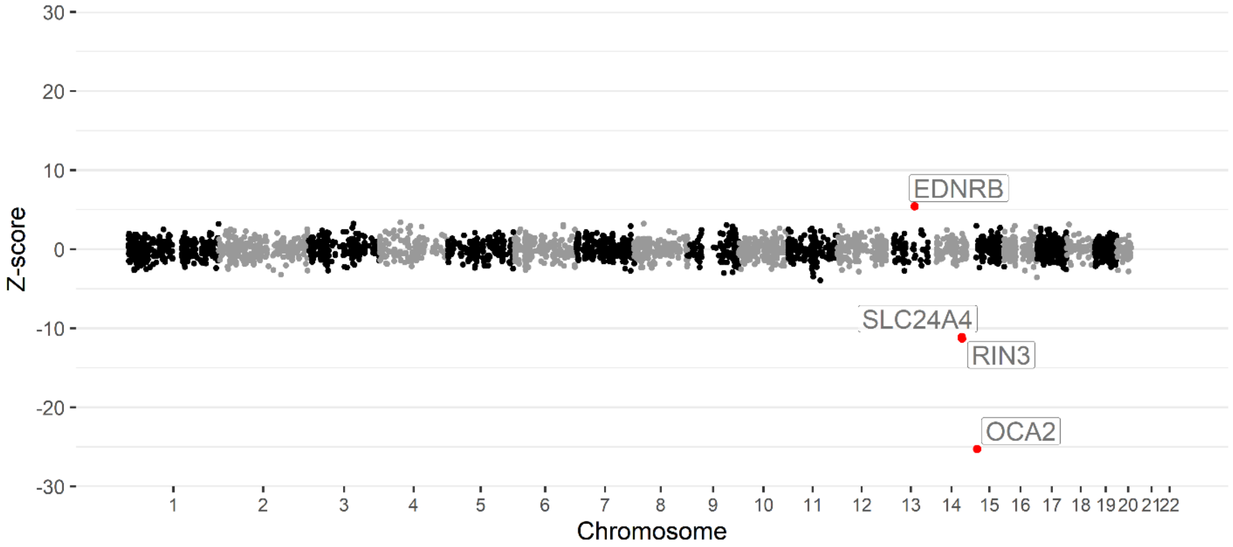

Supplementary Figure 12. Miami plot of the TWAS conducted for blonde hair colour across chromosomes (X-axis) against TWAS Z-scores (Y-axis). Genes that passed the significance threshold (p-value ≤ 4.17e-6) are annotated and highlighted in red.

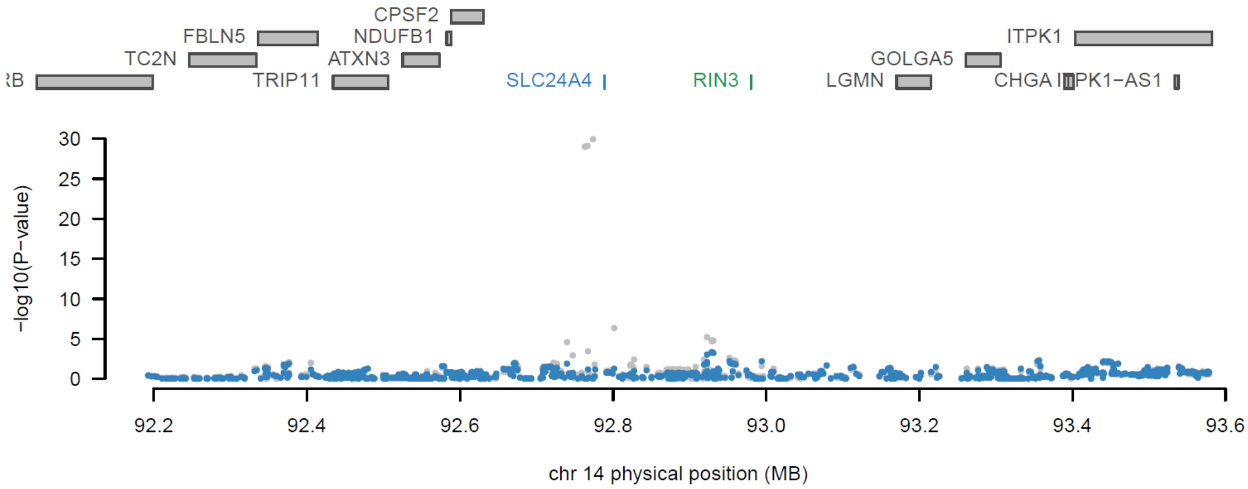

Supplementary Figure 13. Conditional analysis of TWAS analysis of the locus in chromosome 14. The top section shows all the genes in the locus: *SLC24A4* and *RIN3*, in blue and green respectively, and other genes are annotated in grey (genome version hg19). The bottom section illustrates the GWAS original regional plot in grey, and after conditioning for the predicted expression of *RIN3* in blue. The GWAS signal disappears after the conditional analysis, showing that the genes *SLC24A4* and *RIN3* are not independent from each other.

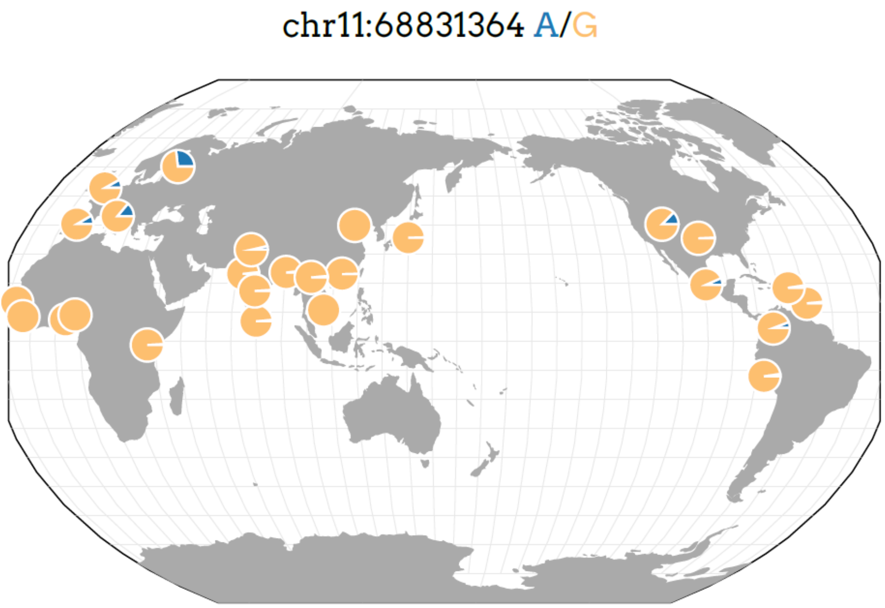

Supplementary Figure 14. Frequency of rs72928978 on *TPCN2* in different world populations from the 1KGP Phase 3, in which the A-allele (associated with blonde hair colour, depicted in blue) is present only in European populations and populations with European ancestry [2].

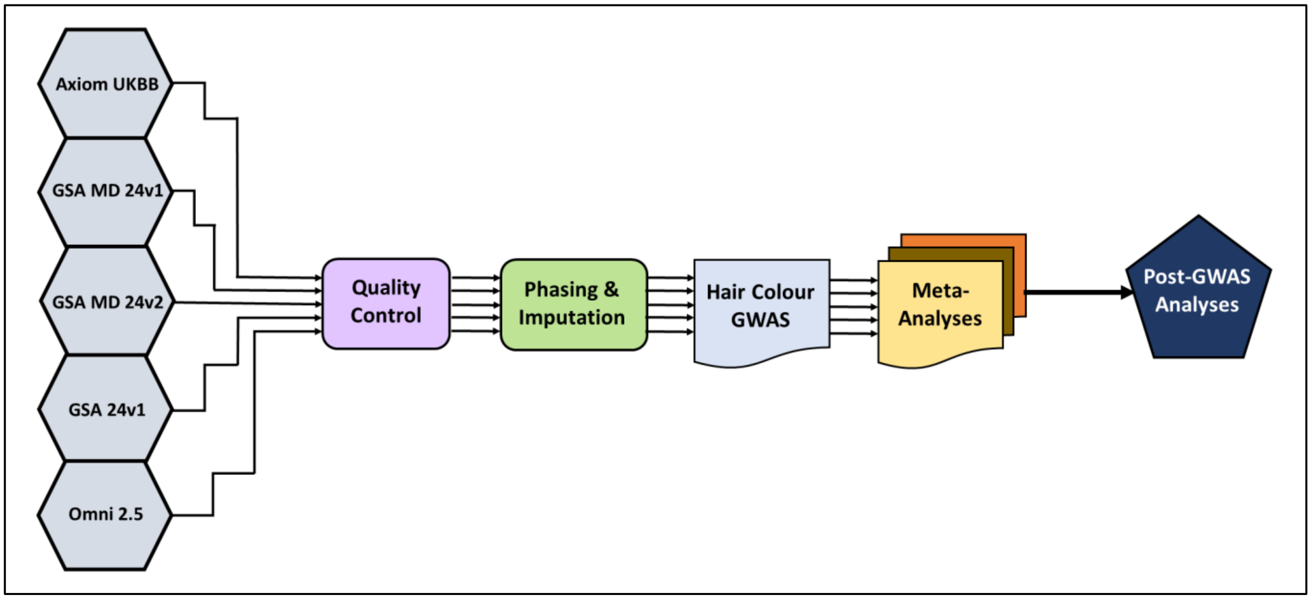

Supplementary Figure 15. Workflow of the overall methodology followed in this study.

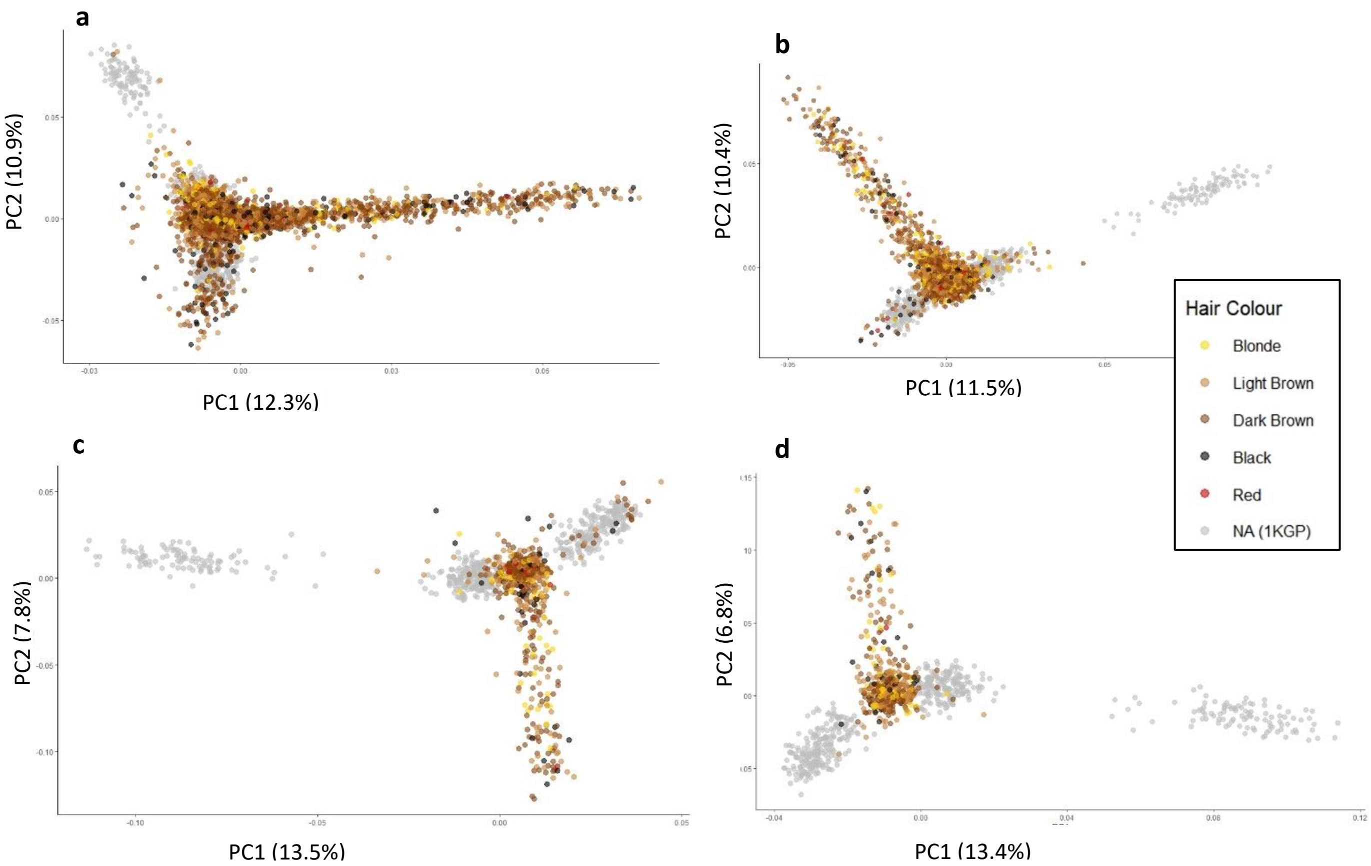

Supplementary Figure 16. Principal Components Analysis of Quebec samples from CanPath (a = GSA 24v1+MDP; b = GSA 24v2+MDP; c = Omni 2.5; d = GSA 24v1) with 502 European samples from the 1000 Genomes Project Phase 3 (CEU, FIN, GBR, IBS, TSI).

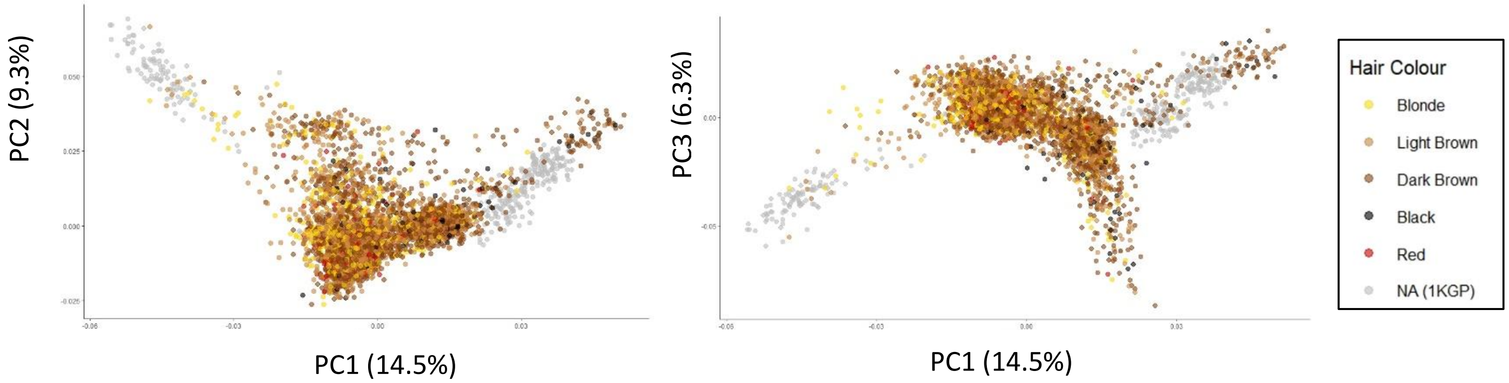

Supplementary Figure 17. Principal Components Analysis of 4,745 samples from CanPath (Atlantic Provinces, Alberta, British Columbia, Quebec and Ontario) with 502 European samples from the 1000 Genomes Project Phase 3 (CEU, FIN, GBR, IBS, TSI). a = PC1 vs. PC2; b = PC1 vs. PC3.

Supplementary References:

1. Higgins, J. P. T. & Thompson, S. G. Quantifying heterogeneity in a meta-analysis. *Stat. Med.* **21**, 1539–1558 (2002).

2. Marcus, J. H. & Novembre, J. Genetics and population analysis Visualizing the geography of genetic variants. **33**, 594–595 (2017).
