# Supplementary material for: A large Canadian cohort provides insights into the genetic architecture of human hair colour
Source: Commun Biol. 2021 Nov 4;4:1253. doi: 10.1038/s42003-021-02764-0 (PMC8568909; doi:10.1038/s42003-021-02764-0)
Supplement: Supplementary file 3 — Description of Additional Supplementary Files [file 42003_2021_2764_MOESM3_ESM.pdf]

Description of Additional Supplementary Files

File name: Supplementary Data

Description:

Supplementary Data 1: Source data of Figure 1: Proportion of hair colour categories by province and by sex.

Supplementary Data 2: Suggestive ($p < 1e-6$) and genome-wide ($p < 1.67e-8$) associated SNPs in the meta-analysis of blonde vs. brown and black hair colour.

Supplementary Data 3: Suggestive ($p < 1e-6$) and genome-wide ($p < 1.67e-8$) associated SNPs in the meta-analysis of brown vs. black hair colour.

Supplementary Data 4: Suggestive ($p < 1e-6$) and genome-wide ($p < 1.67e-8$) associated SNPs in the meta-analysis of red vs. brown and black hair colour.

Supplementary Data 5: Suggestive ($p < 1e-6$) and genome-wide ($p < 5e-8$) associated SNPs in the meta-analysis using a linear mixed model.

Supplementary Data 6: GCTA-COJO results for each hair colour model using a secondary LD reference matrix.

Supplementary Data 7: Candidate causal variants with annotation in the blonde vs. brown and black hair colour model.

Supplementary Data 8: Candidate causal variants with annotation in the brown vs. and black hair colour model.

Supplementary Data 9: Candidate causal variants with annotation in the red vs. brown and black hair colour model.

Supplementary Data 10: Source data of Figure 3: Pathway enrichment analysis with FUMA.
