# Supplementary material for: A large Canadian cohort provides insights into the genetic architecture of human hair colour
Source: Commun Biol. 2021 Nov 4;4:1253. doi: 10.1038/s42003-021-02764-0 (PMC8568909; doi:10.1038/s42003-021-02764-0)
Supplement: Supplementary file 5 — Reporting Summary [file 42003_2021_2764_MOESM5_ESM.pdf]

nature research

Corresponding author(s): Frida Lona-Durazo and Esteban J. Parra

Last updated by author(s): Aug 23, 2021

Reporting Summary

Nature Research wishes to improve the reproducibility of the work that we publish. This form provides structure for consistency and transparency in reporting. For further information on Nature Research policies, see our Editorial Policies and the Editorial Policy Checklist.

Statistics

For all statistical analyses, confirm that the following items are present in the figure legend, table legend, main text, or Methods section.

n/a	Confirmed	
☐	☒	The exact sample size (*n*) for each experimental group/condition, given as a discrete number and unit of measurement
☒	☐	A statement on whether measurements were taken from distinct samples or whether the same sample was measured repeatedly
☐	☒	The statistical test(s) used AND whether they are one- or two-sided<br>*Only common tests should be described solely by name; describe more complex techniques in the Methods section.*
☐	☒	A description of all covariates tested
☐	☒	A description of any assumptions or corrections, such as tests of normality and adjustment for multiple comparisons
☐	☒	A full description of the statistical parameters including central tendency (e.g. means) or other basic estimates (e.g. regression coefficient) AND variation (e.g. standard deviation) or associated estimates of uncertainty (e.g. confidence intervals)
☐	☒	For null hypothesis testing, the test statistic (e.g. *F*, *t*, *r*) with confidence intervals, effect sizes, degrees of freedom and *P* value noted<br>*Give P values as exact values whenever suitable.*
☒	☐	For Bayesian analysis, information on the choice of priors and Markov chain Monte Carlo settings
☒	☐	For hierarchical and complex designs, identification of the appropriate level for tests and full reporting of outcomes
☐	☒	Estimates of effect sizes (e.g. Cohen's *d*, Pearson's *r*), indicating how they were calculated

Our web collection on statistics for biologists contains articles on many of the points above.

Software and code

Policy information about availability of computer code

Data collection	We collected gene and regulatory annotations using SNPNexus v4. No other software was used for data collection.
Data analysis	PLINK v1.9, PLINK v2.0, Sanger Imputation Server, EAGLE v2.0.5, SAIGE, R v3.5.1, qqman and ggplot2 (R packages), METASOFT 2.0.1, GCTA, FINEMAP v1.4, LDstore v2.0, hyprcoloc (R package), FUSION, FUMA, LDSC.

For manuscripts utilizing custom algorithms or software that are central to the research but not yet described in published literature, software must be made available to editors and reviewers. We strongly encourage code deposition in a community repository (e.g. GitHub). See the Nature Research guidelines for submitting code & software for further information.

Data

Policy information about availability of data

All manuscripts must include a data availability statement. This statement should provide the following information, where applicable:

- Accession codes, unique identifiers, or web links for publicly available datasets
- A list of figures that have associated raw data
- A description of any restrictions on data availability

We provide the genome-wide (p ≤ 1.67e-8) and suggestive (p ≤ 1e-6) signals identified in the meta-analyses as a Supplementary Data (Supplementary Data 1-3). The complete summary data of the meta-analyses have been deposited in the GWAS Catalog. Raw genotype or phenotype data from the CanPath cannot be made available due to restrictions imposed by the ethics approval. The genotyping and methylation data of cultured melanocytes are available through the Gene Expression Omnibus (GEO) database with the accession numbers: GSE101771 and GSE166069, respectively.

Field-specific reporting

Please select the one below that is the best fit for your research. If you are not sure, read the appropriate sections before making your selection.

- [x] Life sciences
- [ ] Behavioural & social sciences
- [ ] Ecological, evolutionary & environmental sciences

For a reference copy of the document with all sections, see nature.com/documents/nr-reporting-summary-flat.pdf

Life sciences study design

All studies must disclose on these points even when the disclosure is negative.

Sample size	We included all participants from the CanPath, for which genome-wide genotype data and hair colour data were available (N = 12,741).
Data exclusions	We excluded SNPs with genotyping rate < 95% and those that deviated significantly from Hardy-Weinberg Equilibrium, as well as participants with genotyping rate <95%, second-degree relatives or those who did not cluster with the European sample of the 1000 Genomes Project.
Replication	We followed-up the candidate causal signals of our meta-analyses in recent pigmentation studies.
Randomization	Not applicable, as it is a GWAS meta-analysis.
Blinding	Not relevant for this study, as it is a GWAS meta-analysis.

Reporting for specific materials, systems and methods

We require information from authors about some types of materials, experimental systems and methods used in many studies. Here, indicate whether each material, system or method listed is relevant to your study. If you are not sure if a list item applies to your research, read the appropriate section before selecting a response.

Materials & experimental systems

n/a	Involved in the study	
x		Antibodies
x		Eukaryotic cell lines
x		Palaeontology and archaeology
x		Animals and other organisms
	x	Human research participants
x		Clinical data
x		Dual use research of concern

Methods

n/a	Involved in the study	
x		ChIP-seq
x		Flow cytometry
x		MRI-based neuroimaging

Human research participants

Policy information about studies involving human research participants

Population characteristics	The average age of the CanPath participants was 53 years old, and 53.8% were females. We included both of these data as fixed effects in our GWAS. All genotyped individuals self-reported European ancestry and this was corroborated through PCA with 1000 Genomes Project samples. We also controlled for population substructure in the GWAS using the first 10 eigenvectors from the PCA as fixed effects, and using a genetic relationship matrix as random effect.
Recruitment	Participants were recruited in the CanPath cohort as part of a large national effort in different provinces. Participants were selected to be genotyped if they did not have or have had cancer at the time of selection and if they had self-reported a European ancestry as part of a questionnaire.
Ethics oversight	This study was approved by the University of Toronto Ethics Committee (Human Research Protocol #36429) and data access was granted by the Canadian Partnership for Tomorrow's Health (Application number DAO-034431).

Note that full information on the approval of the study protocol must also be provided in the manuscript.
